# Multimodal Neuroimaging-Informed Clinical Applications in Neuropsychiatric Disorders

**DOI:** 10.3389/fpsyt.2016.00063

**Published:** 2016-04-19

**Authors:** Rafael O’Halloran, Brian H. Kopell, Emma Sprooten, Wayne K. Goodman, Sophia Frangou

**Affiliations:** ^1^Brain Imaging Center, Translational and Molecular Imaging Institute, Icahn School of Medicine at Mount Sinai, New York, NY, USA; ^2^Department of Neurosurgery, Icahn School of Medicine at Mount Sinai, New York, NY, USA; ^3^Department of Neurology, Icahn School of Medicine at Mount Sinai, New York, NY, USA; ^4^Department of Neuroscience, Icahn School of Medicine at Mount Sinai, New York, NY, USA; ^5^Department of Psychiatry, Icahn School of Medicine at Mount Sinai, New York, NY, USA

**Keywords:** deep brain stimulation, machine learning applied to neuroscience, multimodal imaging, individual variability, precision psychiatry

## Abstract

Recent advances in neuroimaging data acquisition and analysis hold the promise to enhance the ability to make diagnostic and prognostic predictions and perform treatment planning in neuropsychiatric disorders. Prior research using a variety of types of neuroimaging techniques has confirmed that neuropsychiatric disorders are associated with dysfunction in anatomical and functional brain circuits. We first discuss current challenges associated with the identification of reliable neuroimaging markers for diagnosis and prognosis in mood disorders and for neurosurgical treatment planning for deep brain stimulation (DBS). We then present data on the use of neuroimaging for the diagnosis and prognosis of mood disorders and for DBS treatment planning. We demonstrate how multivariate analyses of functional activation and connectivity parameters can be used to differentiate patients with bipolar disorder from those with major depressive disorder and non-affective psychosis. We also present data on connectivity parameters that mediate acute treatment response in affective and non-affective psychosis. We then focus on precision mapping of functional connectivity in native space. We describe the benefits of integrating anatomical fiber reconstruction with brain functional parameters and cortical surface measures to derive anatomically informed connectivity metrics within the morphological context of each individual brain. We discuss how this approach may be particularly promising in psychiatry, given the clinical and etiological heterogeneity of the disorders, and particularly in treatment response prediction and planning. Precision mapping of connectivity is essential for DBS. In DBS, treatment electrodes are inserted into positions near key gray matter nodes within the circuits considered relevant to disease expression. However, targeting white matter tracts that underpin connectivity within these circuits may increase treatment efficacy and tolerability therefore relevant for effective treatment. We demonstrate how this approach can be validated in the treatment of Parkinson’s disease by identifying connectivity patterns that can be used as biomarkers for treatment planning and thus refine the traditional approach of DBS planning that uses only gray matter landmarks. Finally, we describe how this approach could be used in planning DBS treatment of psychiatric disorders.

## Introduction

The neuropathology underlying psychiatric disorders is poorly defined, and, consequently, psychiatric nosology is mainly informed by clinical observation. As a result, psychiatric diagnoses are likely heterogeneous, multifaceted, and overlapping in their etiology and pathophysiology (1). This motivates efforts to characterize valid and reliable biological markers of disease expression in order to facilitate early identification and novel treatment discovery. Neuroimaging has already had a transformative role in psychiatry, as it has established that psychiatric disorders are disorder of the brain. Magnetic resonance imaging (MRI) methods, and particularly functional MRI (fMRI), diffusion-weighted, and diffusion tensor imaging (DWI/DTI), have been extensively used to assess alterations in brain functional and structural organization associated with psychiatric disorders (2, 3). Findings from the neuroimaging literature have improved the characterization of the biological underpinnings of psychiatric disorders but have had limited clinical utility, as they are based on group-level inferences that cannot be readily applied to single individuals (4).

The term “third-generation imaging” collectively describes the development of new paradigms in image acquisition and analysis that aim to identify brain markers that can improve diagnostic assessment and prognostic formulations and lead to personalized treatment planning (4). In this article, we highlight the potential of multivariate pattern recognition methods to address areas of diagnostic and prognostic uncertainty in mood disorders, and we then focus on the promise of high-field imaging in leading to identification and targeting of patient-specific neural targets.

## Multivariate Pattern Recognition Methods in Psychiatric Disorders

**Figure 1:**
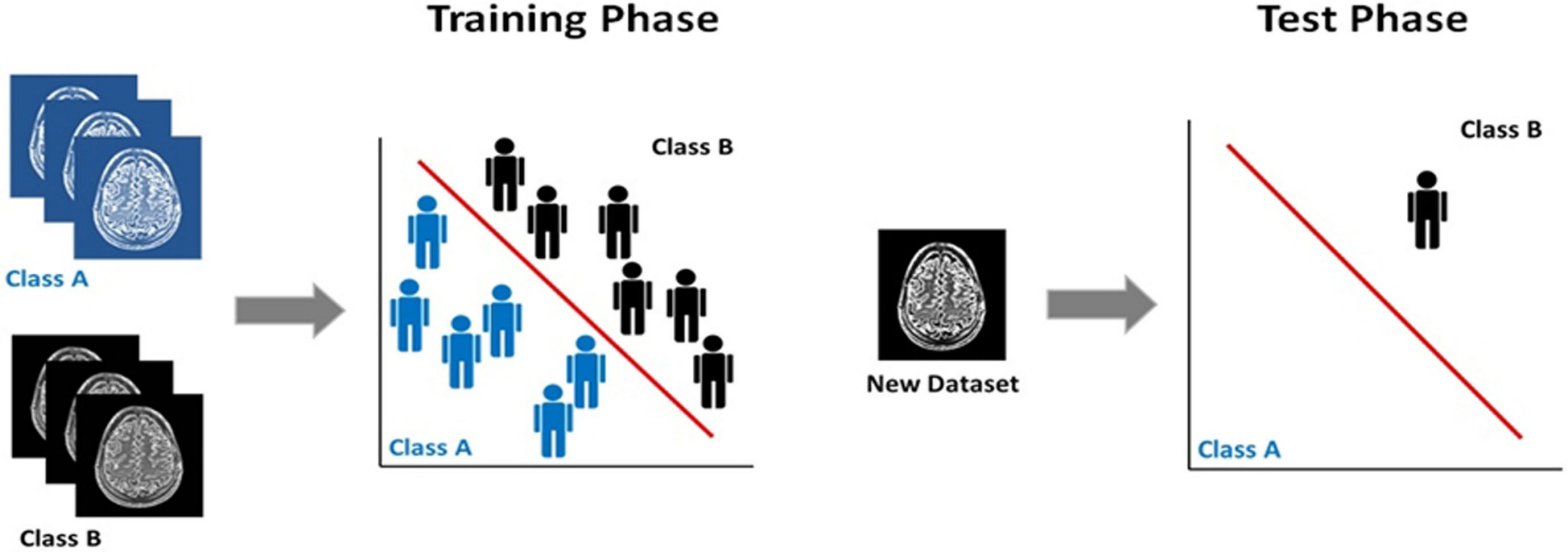
**Supervised classification using multivariate pattern recognition models**. Classification training and test phase: the classifier in initially trained on a set with predefined class labels and the classification rule derived is used to assign class membership to a previously unseen dataset.

The majority of neuroimaging studies in psychiatry use voxel-based statistics (e.g., general linear model), which are biased toward detecting group-level differences that are highly localized in space and linear in nature. However, structural and fMRI data are inherently multivariate since each imaging dataset contains information distributed among the thousands of its constituent voxels. Over the last 5 years, there has been increasing interest in multivariate pattern recognition methods, as these methods can capture potentially useful information embedded in the spatial pattern of the data. Multivariate pattern recognition can be achieved through several statistical models. Regardless of the model, pattern recognition tools rely on computational algorithms to discover regularities in the data, which are then used to derive rules for inferring individual-level characterization (5) (Figure 1). This feature is of translational value, as it is aligned with the person-centered nature of clinical practice. In psychiatric neuroimaging, multivariate pattern recognition methods have been mostly used to classify individuals into discreet categories according to diagnostic status (e.g., patients, healthy controls), prognosis (e.g., converters, non-converters), or treatment response (e.g., treatment responders, non-responders). Frequently used classifiers are support vector machines and Gaussian process classifiers, which use a supervised approach to classification. This means that the algorithm is first trained to identify regularities in the neuroimaging data that discriminate individuals whose status is predefined. For example, the classifier is given imaging data from patients and healthy controls and is trained to generate a classification rule that discriminates the two groups. In the next phase, the test phase, the classifier is presented with a dataset from a previously unseen individual and uses the classification rule to determine the status of this new example. Sensitivity, specificity, and accuracy are the most commonly reported measures of classifier performance in terms of the accuracy of the classification rule in determining the status of previously unseen individual datasets. In the case of binary classifiers, for example, involving patients and controls, sensitivity refers to the proportion of patients (true positives) who are correctly identified as patients, whereas specificity measures the proportion of controls (true negatives) who are correctly identified as controls. The accuracy of the classifier refers to the total proportion of patients and controls that are correctly classified. Furthermore, permutation testing is also employed to determine whether the results of the pattern recognition model deviate significantly from chance. In linear classifiers, voxels can be visualized on the basis of their contribution to classification thus producing discriminative maps (6). The relevant literature in psychiatry has recently been summarized in multiple reviews that provide a comprehensive account of the progress to date and the challenges that still lie ahead (7–10). The field is dominated by studies that sought to discriminate healthy individuals from patients with either schizophrenia (*n* = 51) or major depressive disorder (MDD) (*n* = 31) using structural, diffusion-weighted, and fMRI data; the reported accuracies of these case–control classifiers range between 71 and 96% for schizophrenia and between 61 and 96% for MDD (10).

We focus here on bipolar disorder (BD) where the current evidence base is rather limited despite the fact that BD ranks among the leading causes of disability worldwide across all age groups (11). BD is a mood disorder characterized by episodes of depression and mania and executive function impairments. Our group (12) and other labs (13–17) have tested the value of structural MRI data in discriminating patients with BD from healthy individuals leading to poor (15) or moderate results (12, 16, 17). Investigations using fMRI data from tasks of verbal fluency (18), facial affect processing (19–21), auditory oddball detection (22), and working memory (23) have been more promising, resulting in overall accuracy of approximately 80%. The reported classification accuracies do not appear substantially influenced by the type of task or the neuroimaging features (connectivity measures or whole-brain task-based signal change) used for classification (18–23). This evidence demonstrates the potential utility of pattern recognition models as a diagnostic aid in BD, although it does not address the complexity of diagnostic challenges in clinical settings.

It could be argued that neuroimaging-based tools may prove more useful in situations where clinical assessment and observation alone are known to be insufficient. The diagnostic and prognostic outcome of first-degree relatives of patients with BD presents with several such situations of genuine clinical uncertainty. We therefore tested the hypothesis that neuroimaging, coupled with pattern recognition analyses, might assist in the evaluation of individuals at risk of BD by virtue of a positive family history for this disorder. Genetic proximity to patients remains the most significant predictor of morbidity that is not limited to increased risk for BD but includes other adverse outcomes, most commonly MDD. The respective morbidity risks in first-degree relatives of bipolar patients generally range from 4 to 6% for BD and 11 to 18% for MDD (24). There are two key areas of clinical uncertainty in assessing first-degree relatives of bipolar patients. The first refers to the differential diagnosis of BD from MDD in these individuals. Differentiating these two disorders is generally challenging (25), because the clinical presentation of both disorders is dominated by depressive symptoms (26). There are several demographic and clinical features that are more indicative of bipolar depression but, at the level of the individual patient, depressive episodes arising in the context of BD do not differ substantially from those seen in MDD (27–29). Furthermore, depressive episodes generally precede the onset of the first manic episode by several years (30). It is therefore rather common for individuals with BD to present with depression and to be incorrectly diagnosed as having MDD. The consequences of misdiagnosis include potential worsening of the illness course and greater psychosocial disability (25). In this scenario, it would be helpful to have a diagnostic tool that could differentiate BD from MDD in individuals with positive family history of bipolarity. The second challenge refers to the accurate risk stratification of individuals with a positive family history of BD. As discussed first-degree relatives of patients, as a group, have a higher risk than the general population for affective morbidity (24, 31). However, a substantial number of relatives, up to 60%, may remain free of psychopathology. Correctly identifying individuals who are very unlikely to present with a psychiatric disorder is critical for developing a neuroscience-informed framework for targeted early intervention.

In order to address these issues, we obtained working memory task-related fMRI data from 120 demographically and IQ matched participants consisting of 30 patients with BD-type I (15 men and 15 women, mean age = 34.7 years, SD = 7.7 years), 30 of their first-degree relatives diagnosed with MDD (16 men, 15 women, mean age = 32.9 years, SD = 9.9 years), 30 psychiatrically healthy first-degree relatives (14 men, 16 women, mean age = 35.3 years, SD = 5.6 years), and 30 unrelated healthy controls (15 men, 15 women, mean age = 33.4 years, SD = 11.6 years). Only six participants were related to each other. Participants with BD or MDD were in symptomatic remission at the time of scanning, defined as a total score of <7 in the Hamilton Depression Rating Scale and in the Young Mania Rating Scale. Patients with BD were prescribed atypical antipsychotics (*n* = 21), antiepileptics (*n* = 8), and lithium (*n* = 14), as monotherapy (*n* = 18) or combination therapy (*n* = 12). Three relatives with MDD were on selective serotonin reuptake inhibitors.

Images were acquired using a 1.5-T GE Neuro-optimized Signa MR system (General Electric, Milwaukee, WI, USA) fitted with 40 mT/m high speed gradients. The MRI protocol included a total of 180 T2*-weighted MR brain volumes depicting blood–oxygen level dependent (BOLD) contrast acquired at each of 36 near-axial planes parallel to the inter-commissural plane; repetition time (TR) = 3000 ms, echo time (TE) = 40 ms, slice thickness = 3 mm, voxel dimensions = 3.75 mm × 3.75 mm × 3.30 mm, interslice gap = 0.3 mm, matrix size = 64 × 64, and flip angle = 9°. During the same session, a high-resolution, T1-weighted structural image was acquired in the axial plane [inversion recovery prepared, spoiled gradient-echo sequence; inversion time (TI) = 450 ms, TR = 18 ms, TE = 5.1 ms, slice thickness = 1.5 mm, voxel dimensions = 0.9375 mm × 0.9375 mm × 1.5 mm, matrix size 256 × 192, field of view (FOV) = 240 mm × 180 mm, flip angle = 20°, and number of excitations = 1]. Task-related fMRI data were obtained using the typical letter-based 3-back task in a block design with the 0-back condition as sensorimotor control. We chose the 3-back contrast as in our view, and it represents a selection of an enriched feature for pattern recognition analysis as individual differences in activation patterns are more reliably observed in more demanding task conditions (32). The images were realigned, normalized to the Montreal Neurological Institute (MNI) template, smoothed (using an 8-mm Gaussian kernel), and analyzed using a conventional general linear model. All fMRI data processing and analyses were implemented Statistical Parametric Mapping (SPM8)^1^. Performance was evaluated in terms of reaction time to target letters and accuracy (% correct responses). Accuracy was 69.8 (16.7) in patients with BD, 73.4 (17.2) in relatives with MDD, 88.5 (14.3) in healthy relatives, and 73.2 (12.4) in healthy controls. The only significant differences concerned the healthy relatives who outperformed all other groups (all *p* < 0.01). Further details of the sample and the paradigm can be found in the original studies (21, 23, 33–48).

Binary Gaussian process classifiers (49) using whole-brain individual beta maps for the contrast of 3-back vs. 0-back were implemented in the Pattern Recognition for Neuroimaging Toolbox (PRoNTo)^2^ in order to determine their usefulness in differentiating patients with BD (a) from healthy individuals, (b) from relatives with MDD, and (c) from psychiatrically healthy relatives. Each of these classifiers was trained using a leave-two-out cross-validation iteration, whereby model is repeatedly refit leaving out a pair of observation from each group and then used to derive a prediction for the left-out observations. For each trial, we thresholded the probabilistic predictions at 0.5 to convert the probabilistic predictions to class labels allowing the sensitivity and specificity of classification to be computed over all trials. The statistical significance of each classifier was determined by permutation testing by repeatedly retraining the classifier after permuting the class labels (1000 permutations). A *p*-value for classification accuracy was computed by counting the number of permutations for which the permuted accuracy was equal or greater than the true accuracy (obtained with non-permuted labels), then dividing by 1000. Each classifier yields a discrimination map with the spatial distribution of voxels that contributed to the discrimination function.

Patients with BD were discriminated from unrelated healthy controls with a sensitivity (true positives for BD) of 84.6%, specificity (true negatives for unrelated controls) of 92.3%, and overall accuracy of 83.5% (*p* = 0.001). The largest clusters discriminating patients with BD from unrelated controls were located in the prefrontal cortex (encompassing the left inferior, middle, and superior frontal gyrus and in the superior parietal lobule). This finding is in line with results reported in other samples of patients with BD using classifiers based on different activation tasks (18–23).

**Figure 2:**
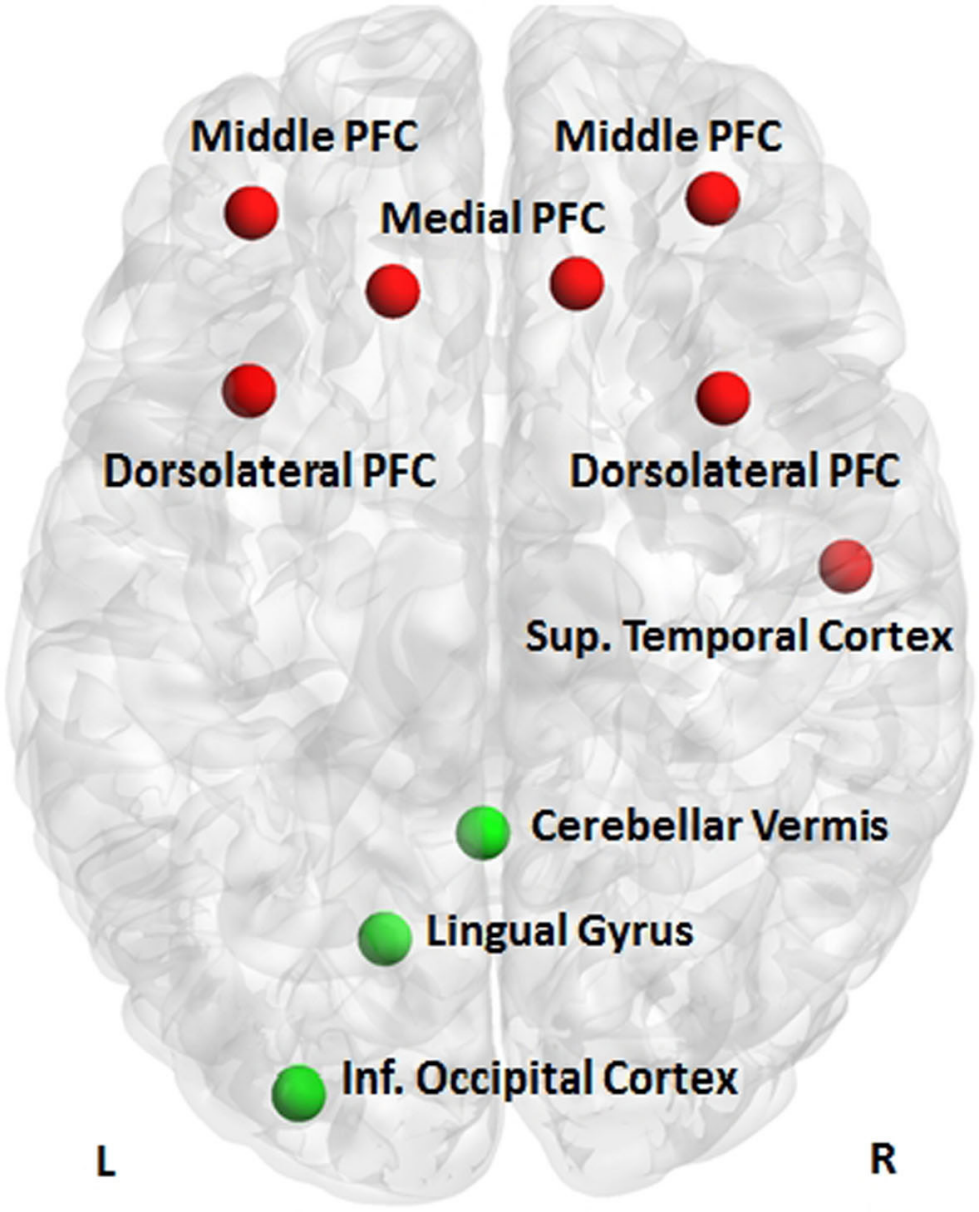
**Discriminative clusters**. Clusters discriminating patients with bipolar disorder from their relatives with major depressive disorder (red) or their healthy relatives (green); PFC, prefrontal cortex.

The Gaussian process classifier discriminated patients with BD from their MDD relatives with a sensitivity (true positives for patients with BD) of 53.9%, specificity (true negative for relatives with MDD) of 94.5%, and overall classification accuracy of 73.1% (*p* = 0.001). The largest discriminating clusters were located in the left superior frontal gyrus, right middle frontal gyrus, bilaterally in the middle/superior frontal gyrus, and the right temporal lobe (Figure 2). Previous studies have shown that different task-based fMRI classifiers can discriminate patients with BD from unselected patients with MDD with classification accuracy of approximately 80% (10). The present study shows that similar classification accuracy can be achieved even when patients have family history of BD. Importantly, patients with BD and their relatives with MDD could be classified with high specificity. This opens the possibility of excluding the BD, with a very high level of confidence after a 10-min brain scan, when assessing individuals with MDD at high risk for BD by virtue of a positive family history. Clinical application will require replication in different samples and settings and in more diverse clinical populations. Nevertheless, these findings demonstrate the potential value of neuroimaging in assisting in situations of clinical uncertainty.

Patients with BD could be differentiated from their healthy relatives with a sensitivity (true positives for patients with BD) of 72.7%, specificity (true negative for healthy relatives) of 90.9%, and overall classification accuracy of 81.8% (*p* = 0.004). The largest discriminating clusters were located in the lingual/inferior occipital gyrus and the cerebellum on the left (Figure 2). The high specificity of the classifier denotes that 90% of high-risk individuals unlikely to convert to BD will be correctly identified. Because of the relatively low sensitivity, some true positives may be missed. The ability to provide personalized risk estimates in individuals at familial risk for BD is essential in designing targeted and cost-effective intervention services and in preventing unnecessary treatment, concern, and self-stigmatization in those unlikely to convert to BD. These results are very encouraging and could potentially inform early intervention services, where positive family history is a key criterion of risk and possible service inclusion (31). At-risk mental states are “pluripotential” as family history of a psychiatric disorder is associated with increased risk for multiple adverse health outcomes. Hence, identifying those unlikely to become unwell may be a more sensible strategy than trying to identify “converters” to a specific diagnosis. The clusters contributing to the correct categorization of healthy relatives in this study show biological plausibility, as previous reports have shown that the resilient relatives of patients with BD show increased cerebral volume (34) and greater occipital connectivity (49) when compared to patients or unrelated healthy controls.

The data that we present here demonstrate the promise of pattern recognition models, but there are many challenges to overcome before these models are ready for widespread clinical use. A full review is outside the scope of this article, but key challenges involve testing the generalizability of the results in different samples and across different research centers. The issue of medication contributing to the classifier performance cannot be fully accounted or modeled and will require re-evaluation in drug-free samples. A further challenge is providing appropriate training to clinicians and information to the public to deal with the probabilistic nature of machine learning predictions.

An additional challenge relates to individual variability at the level of brain organization. The development of methodological innovations to improve precision in measuring brain phenotypes will greatly assist in moving the field forward. In the next sections, we describe novel approaches designed to capture individual variability thus improving the translational potential of neuroimaging.

## Precision Mapping of Structural and Functional Connectivity

**Figure 3:**
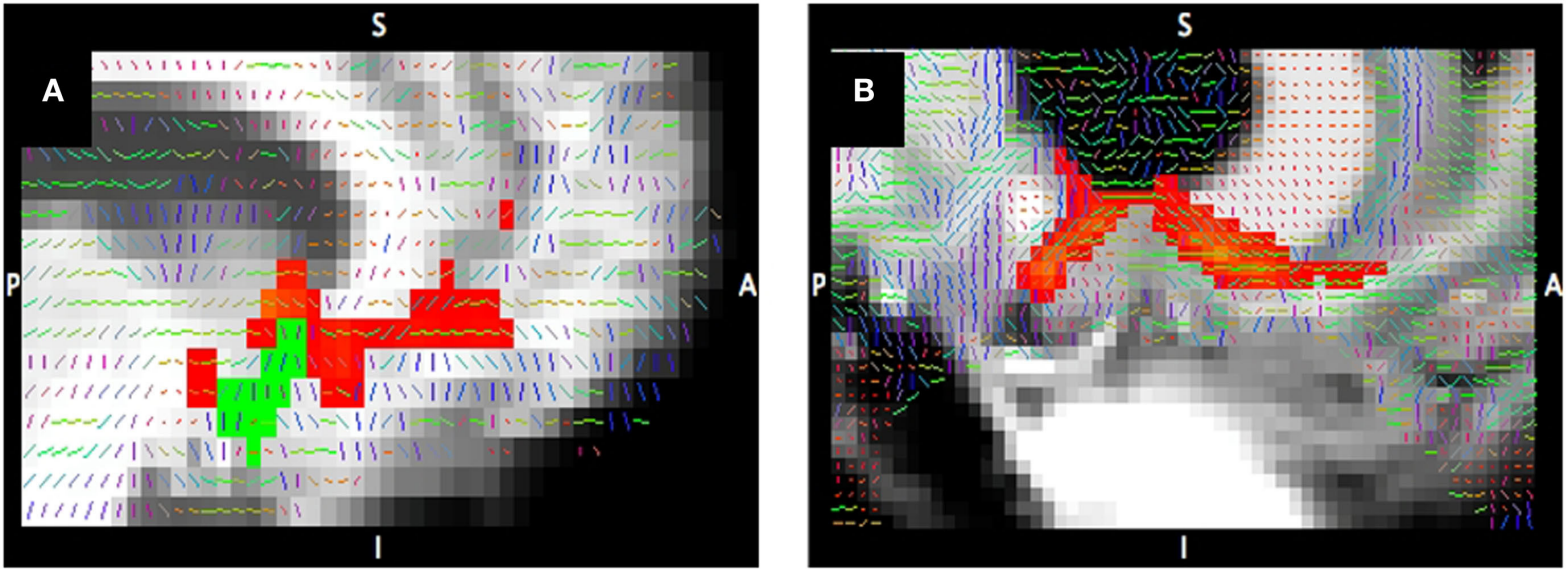
**Tractography at 3-T compared to 7-T**. Tracts connecting the nucleus accumbens (shown in green) to the ventral prefrontal cortex from diffusion-weighted imaging at 3-T **(A)** and 7-T **(B)**. Tract probability map >1% of streamlines (red) corrected for distance; primary diffusion direction overlaid on T1-weighted image.

The increasing availability of high-field MRI scanners, improvements in susceptibility correction methods, and advances in sequence developments enable the *in vivo* investigation of the human brain at higher resolution and higher signal-to-noise ratio than ever before. We illustrate the benefits of high-field imaging using data obtained with DWI/DTI (50), a technique that yields measures of water diffusion within the brain. One DTI-derived measure is fractional anisotropy (FA) that reflects the relative degree to which water diffusion is not evenly restricted in all directions; FA is elevated in areas with high density of white matter tracts due to diffusion being more restricted perpendicular to tracts than along the tracts. Figure 3 provides a visual comparison of FA maps (Figure 3, gray scale images) with the preferred direction of diffusion (Figure 3, colored lines) derived from a 7-T compared to a 3-T diffusion-weighted scan. This demonstrates the improvement in the characterization of the anterior limb of the internal capsule (ALIC) and of the boundary between white matter and cortical gray matter with 7-T compared to 3-T.

A consequence of increased resolution is that inter-individual variability in local morphometry and structure–function correspondence becomes more apparent. Individual differences exist in cortical folding as well as in the relationship of cortical curvature and functional localization (51, 52). However, current standard procedures for functional and anatomical analyses rely on normalization of individual brains to common templates based on stereotaxic coordinates and macro-anatomical landmarks. These procedures do not optimally take into account these individual differences, which can lead to misalignment of neuronal activation between subjects (53). As scan resolution increases, allowing for incrementally fine-grained localization of brain function to cortical gyri and sulci within individual brains, the loss of precision due to normalization paradigms becomes more pronounced. Here, we introduce the concept of “precision mapping,” an approach that addresses this issue by integrating multiple imaging modalities in a single-subject-centered analysis to identify functionally specialized regions in a way does not require normalization to standard templates.

**Figure 4:**
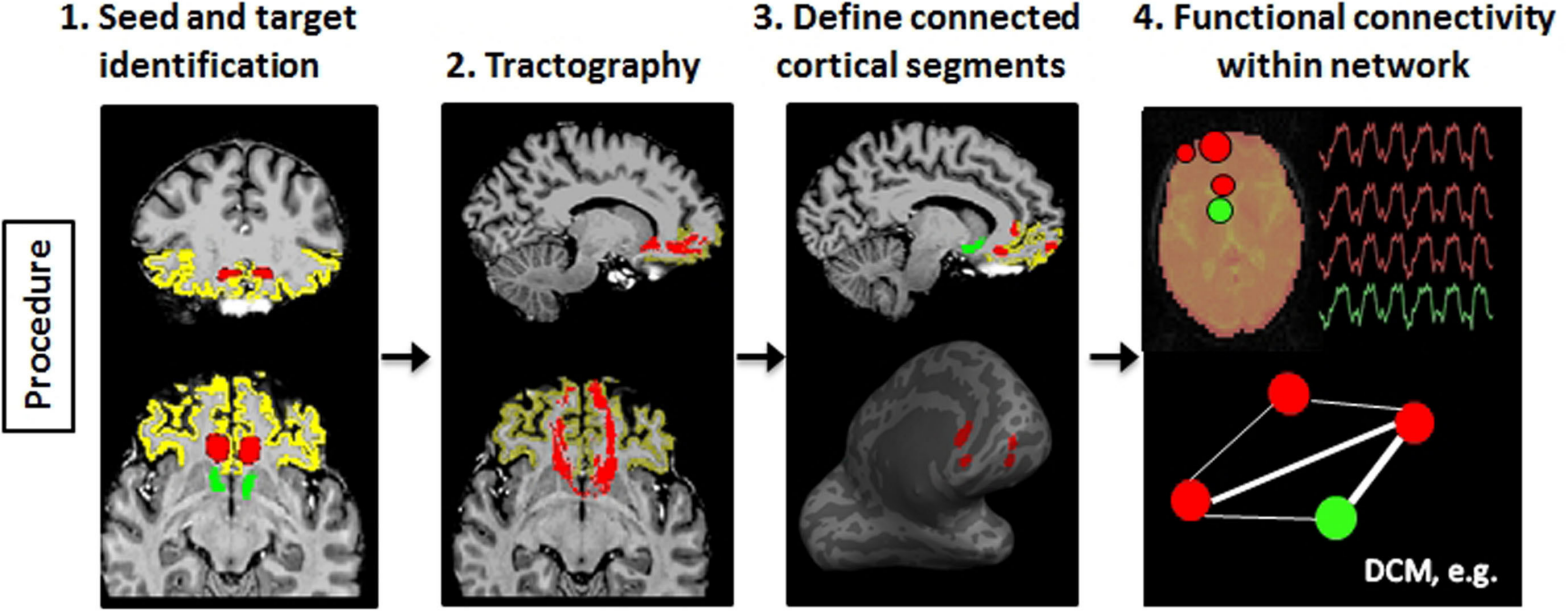
**Schematic illustration of the processes involved in precision mapping**. Step 1: seeds (red) and targets (vPFC: yellow; nucleus accumbens: green) are identified in native space. Step 2: probabilistic tractography is performed. The tract is binarized at 1% of the maximum value (red). Step 3: the segments (red) of the vPFC that is connected to the nucleus accumbens are identified and transformed to the cortical surface in order to include the entire depth of the cortex. Step 4: the cortical segments and the nucleus accumbens segmentation are transformed to the native functional MRI space, and BOLD time series are extracted to perform functional connectivity or dynamic causal modeling (DCM) analysis of the tractography-defined network.

To illustrate the usefulness of precision mapping, we focus on the connectivity profiles of the nucleus accumbens (NAc) and the ventral prefrontal cortex (vPFC). Precision mapping involves the following four steps. First, white matter seeds and gray matter targets were determined in native space on an anatomical T1-weighted scan (Figure 4, step 1). The NAc and the vPFC were defined using Freesurfer^3^ segmentation. For the vPFC, multiple Freesurfer regions were combined, and a superior and posterior boundary was identified in MNI coordinate space and transformed to the individual’s native space. White matter seeds were selected in MNI space, based on the white matter anterior to the NAc and knowledge from tracer studies (54, 55), and transformed to the individual diffusion space. Second, probabilistic tractography was performed using probtrackX2 in FSL (56) (Figure 4, step 2). Third, the endpoints of the tractography were used to identify subregions within the vPFC that is anatomically connected to the NAc, and projected onto the Freesurfer cortical surface to include the full depth of the cortical ribbon (Figure 4, step 3). Fourth, the tractography-determined vPFC subregions and the corresponding subcortical targets were affine transformed to the fMRI space and used for functional connectivity analyses (Figure 4, step 4). The MNI space is only used for the identification of seed regions as a starting point for tractography, but all further processing is completely template independent and therefore individual specific. Thus, the defined network regions can vary in exact location and shape depending on the morphology of the tracts and the cortical surface within each individual.

There are three critical advantages of precision mapping for psychiatric research and treatment development:

(a) Retention of the individual variation: precision mapping does not depend on common neuroanatomical templates or standard atlases. All analyses are performed in each individual’s native space, and affine transformations are only applied to coregister the volumes across the anatomical, diffusion, and functional acquisitions.
(b) Optimized identification of functionally homologous regions: precision mapping defines functionally homologous regions by relying on regional anatomical “connectivity fingerprints” (57), which closely correspond to regional functional specialization at very fine-grained scales in the human brain, the animal brain, and at the neuronal level *in vitro* (58–60). This approach thus enables the identification of functionally homologous regions across individuals, without requiring stereotaxic uniformity of functional organization.
(c) Improved alignment of structural and functional connectivity. Precision mapping allows the identification of individual-specific direct anatomical connections between brain regions of interest. This yields a more complete picture of the functional interactions between regions compared to standard group-based functional connectivity analyses.

**Figure 5:**
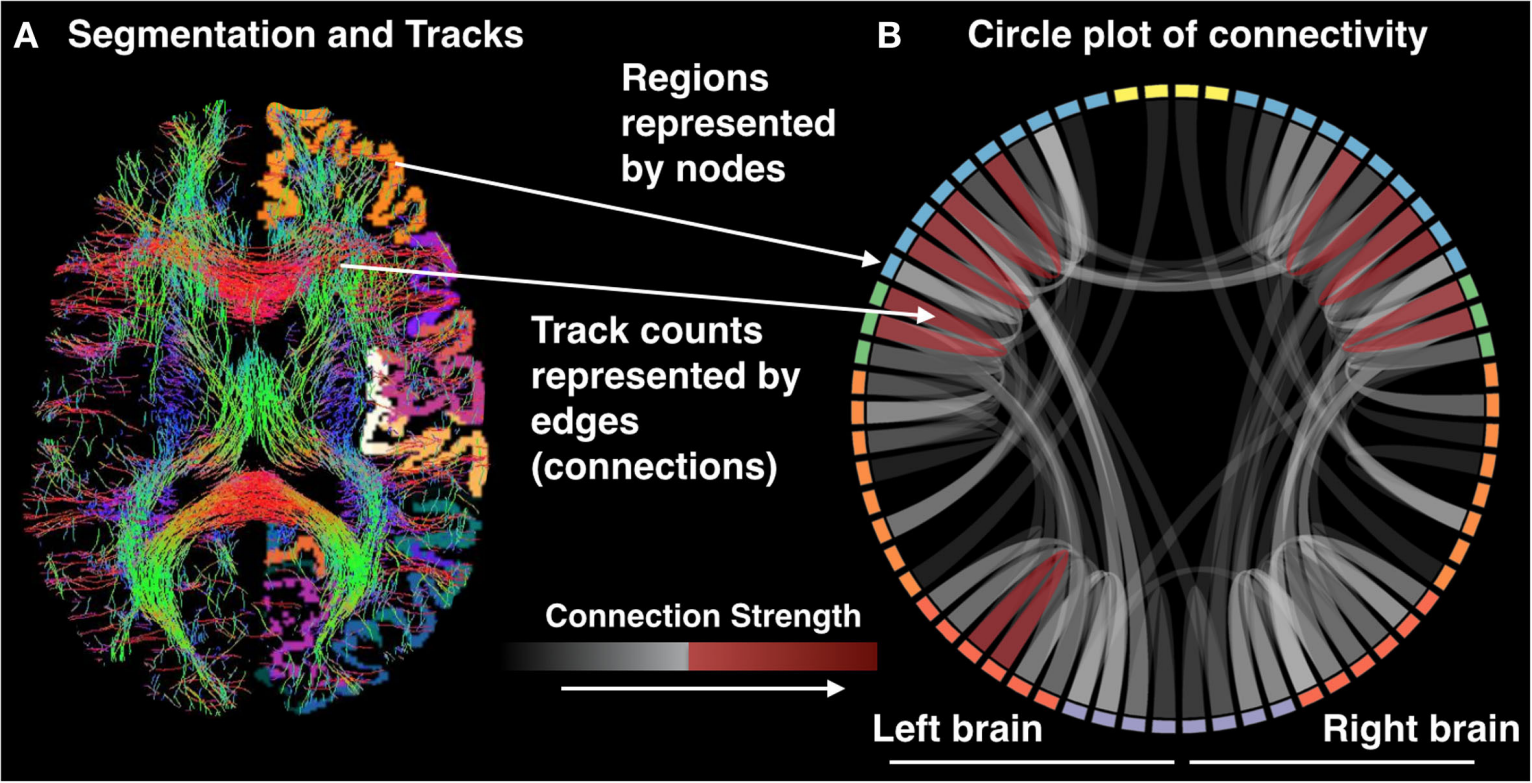
**(A)** Segmentation and tracks and **(B)** circle plot of connectivity.

The multimodal nature of precision mapping is ideally suited to investigate multiple potential causes of network dysfunction within and across individual patients. Qualitative inspection of the data may be on occasion sufficient to identify unusual connectivity segments and can be supplemented by quantitative analyses. Given a large enough sample, the extracted metrics of functional connectivity, anatomical connectivity, and gray matter density may be used to uncover dimensions or different types of network dysconnectivity or (dys)function within patient populations, as well as their variability healthy individuals (Figure 5). Precision mapping is a first step on a new route to clinically translational neuroimaging that can yield an understanding of the nature of brain network pathology in individual patients and its variability between patients.

## Precision Targeting of Patient-Specific Target Networks for Treatment

In neurological disorders, neuroimaging has enabled the successful application of neurosurgical treatments particularly deep brain stimulation (DBS). DBS has been approved by the Food and Drug Administration (FDA) under a humanitarian device exemption for the treatment of movement disorders, such as Parkinson’s disease (PD), dystonia, and essential tremor. Although medications can be remarkably effective at controlling symptoms, DBS is the currently the most effective option to control symptoms and increase quality of life for patients who are refractory (61, 62). The improved efficacy of DBS relies on the targeting of the circuits involved in PD that connect key subcortical nuclei and particularly the globus pallidus interna (GPi), the subthalamic nucleus (STN), the caudal zona incerta (cZi), the red nucleus, and the substantia nigra (61–64). As such, the success of the surgery depends critically on the exact location of the implant in relation to these key brain structures. Visualization of the target circuitry is commonly achieved by sensitizing the MRI signal to the presence of either myelin or iron. Myelin provides contrast between gray and white matter, whereas iron (accumulated in dopaminergic neurons) provides contrast in the basal ganglia and key nuclei of interest in DBS. In conventional DBS, planning lead placement is determined in reference to structures delineated by their myelin and iron content. Although relatively high success rates are achieved by this method, there are cases in which outcomes are suboptimal. A potential explanation for this is that current techniques fail to fully characterize the white matter, and the tissue is composed of highly myelinated axons. Axons are of high relevance to DBS, because they are the most sensitive element of the neuron to the stimulation (65–68). The white matter forms the structural scaffolding of all brain networks. Thus, local stimulation of a key white matter tract with DBS can be thought of as affecting a network whose edges are composed of the fibers of passage around the active electrode. The efficacy of DBS may therefore depend on targeting those white matter tracts that connect and can therefore modulate activity in multiple key multiple regions of disease-related circuits. Direct empirical support for the role of local stimulation has recently been provided in a mouse model of PD using optogenetics and solid-state optics (69). In this model, direct selective stimulation of axonal afferents to the SNT was associated with therapeutic response. Therefore, precision targeting for white matter tracts is important for DBS in humans. White matter tractography was introduced to the field of DBS about a decade ago but has yet to be widely adopted as shown in a recent review that identified only 15 studies with just 66 patients (69). Accumulating evidence, however, suggests that tractography may prove an essential part of treatment planning as the efficacy of DBS is closely associated with the accuracy of lead placement in relation to the target brain regions. For example, Coenen et al. (70) reported that treatment efficacy in patients with PD, essential tremor, and myoclonus dystonia was associated with lead placements close to or within the dentatorubrothalamic tract (DRT). MDD offers another example where variability in DBS outcome has been closely associated with target selection; recent evidence suggests that DBS treatment success may critically depend on electrode placement in the confluence of the uncinate faciculus, forceps, and cingulum bundle (71). Hartmann and colleagues (72) used innovative computational tractography-based activation models to determine the network effects of DBS targets to the NAc and ALIC in patients with obsessive compulsive disorder (OCD). They showed that therapeutic response in OCD patients show tract selectivity. Similar findings in OCD have also been reported by Makris and colleagues (73). Identification of the target tract for DBS implantation is further complicated by the intricate connectivity profiles of many subcortical DBS targets as is the case with GPi, for example, whose connectivity pattern varies along the dorsal to ventral dimension with dorsal GPi regions being more connected to motor regions (74, 75). Precision targeting is therefore essential for the correct identification of the white matter DBS targets.

We focus first on PD because the brain networks involved in disease expression are well-characterized and are known to involve the striatum, pallidum, and midbrain. Nevertheless, at the level of the individual patient, both target network selection and optimal electrode placement remain a challenge. The reason for this may lie in the unique white matter anatomy of each patient and the fact that these deep, centrally located nodes of the network are highly connected “hubs” that serve many functions (76).

In order to explain the efficacy and side effects of DBS, we propose to establish connectivity profiles associated with three common targets for the treatment of PD, namely, the cZI, STN, and GPi (60–63). To do this, the connectivity profiles 17 PD patients who underwent bilateral DBS implantation (5 patients with cZI, 7 patients with STN, and 5 patients with GPi targets) were computed and compared. All patients provided informed consent in accordance with a protocol approved by our institutional review board. All patients were imaged preoperatively with MRI under general anesthesia on a 3-T GE MRI magnet (Discovery 750, GE Healthcare, Waukesha, WI, USA) with a 8-channel receive-only head coil (*Invivo* Corp., Gainesville, FL, USA). The MRI protocol included, a T1-weighted sequence for identifying structure and segmentation, DWI for white matter characterization, quantitative susceptibility mapping (77) to identify iron-rich deep brain nuclei, and contrast-enhanced angiography to identify vessels to be avoided during surgical planning. DWI was performed using a dual spin echo sequence with 60 independent diffusion-encoding directions (*b* = 1000 s/mm^2^) and 5 unweighted images, with in-plane resolution of 2 mm × 2 mm × 3 mm over 61 slices, TR/TE = 7200/82 ms. T1-weighted imaging was performed using an inversion-prepared fast spoiled gradient echo sequence (BRAVO FSPGR) with FOV = 24 cm, resolution 1 mm × 1 mm × 1.2 mm over 164 slices, TR/TE/TI = 8.1/3.1/450 ms, and flip angle 10°. On the day of each surgery (surgeries for left and right implants performed on different days), patients were placed in a stereotactic frame (Leksell G Stereotactic Headframe, Elekta, Stockholm, Sweden) and underwent computed tomography (CT) imaging with 280 mAs, 120 kVP, 0.6 mm × 0.6 mm × 1 mm spatial resolution, 1000 ms exposure time, on a clinical CT system (Sensation Cardiac 64, Siemens, Forcheim, Germany). Postoperative CT images were acquired after the removal of the stereotactic frame.

**Figure 6:**
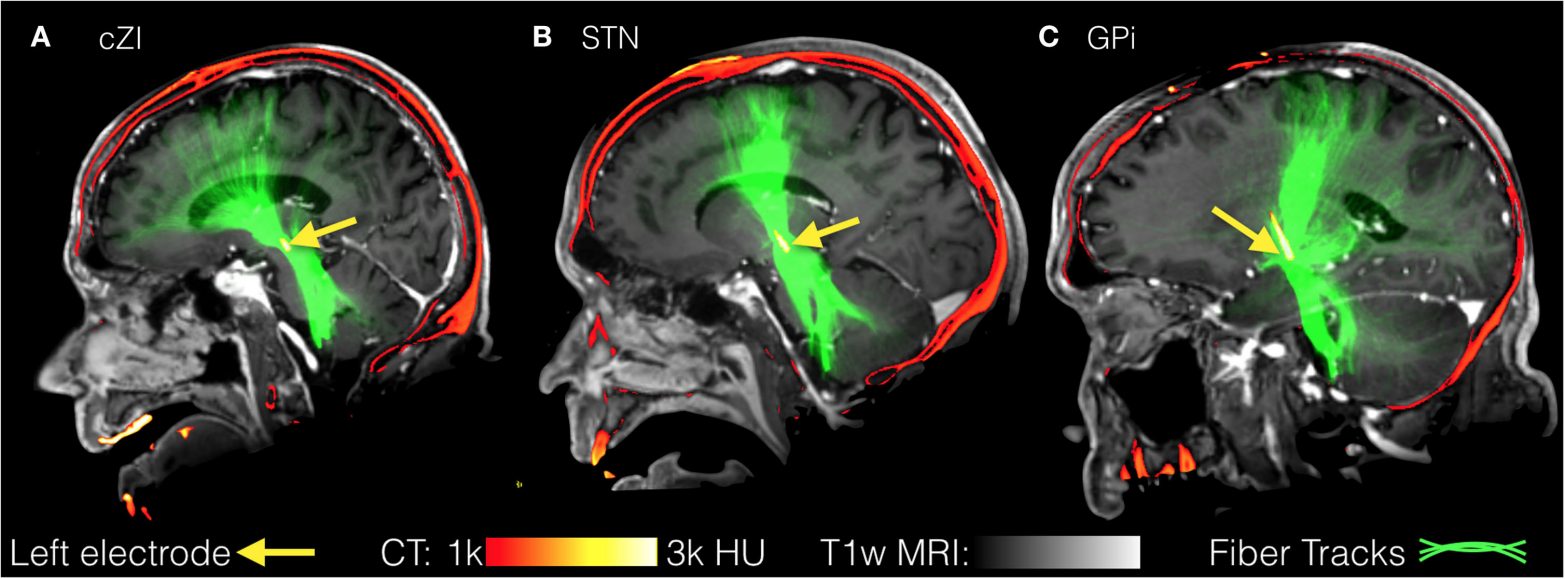
**Fiber tracks from three separate patients with electrodes targeting either the caudal zona incerta (cZi) (A), the subthalamic nucleus (STN) (B), or the globus pallidus interna (Gpi) (C)**. These sagittal views show the fiber tracks that pass through a 3-mm sphere centered on the active electrode and may represent fibers of passage most affected by deep brain stimulation (DBS).

**Figure 7:**
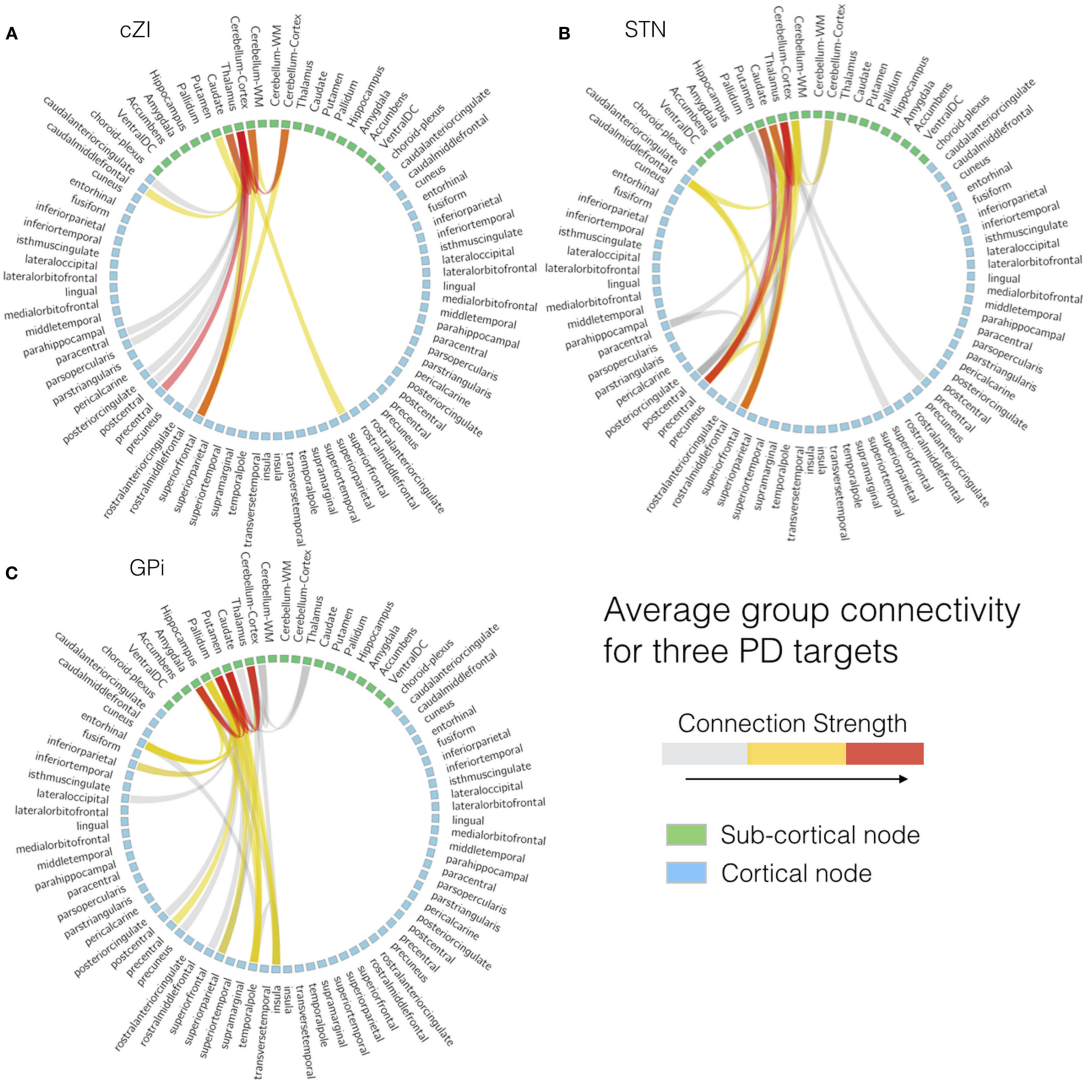
**Individual patient connectivity plots based on electrode location**. Structural connectivity plots from three separate patients with electrodes targeting either **(A)** the caudal zona incerta (cZi), **(B)** the subthalamic nucleus (STN), or **(C)** the globus pallidus interna (Gpi).

All images were first registered to the first preoperative CT scan using FLIRT (FMRIB Linear Image Registration Tool)^4^ and a mutual information cost function. The electrodes were automatically segmented from the second postoperative CT scan (after both electrodes were implanted) using software written in-house in MATLAB (Mathworks, Natick, MA, USA). The segmentation consisted of using a brain mask derived from the structural T1-weighted image to mask out regions outside the brain, then thresholding at 2000 Houndsfield units to obtain only the electrodes. After segmentation, the right and left leads were separately fit to a model of the specific implant (Medtronic 3389 or 3387) to obtain segmentations of the implants including each of the four electrodes and body of the implant. From the segmentation of the electrodes, the centroid of each electrode could be determined. In the structural MR dataset, cortical reconstruction and volumetric segmentation were performed with Freesurfer. The SPGRE image was used as input to Freesurfer. DWI data were corrected for Eddy-current distortions using Eddy_correct in FSL and fit to the preoperative CT using and affine transform. Fiber tracking was performed using the MRtrix package^5^. Constrained spherical deconvolution (78, 79) was performed on preprocessed images to obtain fiber orientation distributions. We used a anatomically constrained probabilistic tractography algorithm (iFOD2) (80) seeded from 3 mm spheres drawn around the centroid of each of the four electrodes, for both the left and right implants, to determine the connectivity pattern of each seed to cortical and subcortical regions using the cortical parcelation and subcortical segmentation algorithms (aparc + aseg) in Freesurfer. Figure 6 shows fiber tracks from three separate patients with electrodes targeting either cZI, STN, or GPi. These sagittal views show the fiber tracks that pass through the 3-mm sphere centered on the active electrode (cathode) and thus can be interpreted as the fibers of passage most affected by DBS. The tracks from all three targets share some common features as they connect with subcortical regions in the brainstem and cerebellum and with cortical regions within superior prefrontal cortex. We then averaged connectivity matrices from the tracks originating from the active electrode separately for each patient group determined by the DBS target (cZI, STN, and GPi). We focused on the electrode associated with better efficacy and tolerability. Group connectivity plots (Figure 7) demonstrate clearly the differences between the three targets. Notably, the cZI and STN targets had strong connections to the contralateral cerebellar cortex that likely encompasses the DRT, as it has a decussation and connects to the contralateral dentate nucleus. Strong connections to the ipsilateral superior frontal cortex may be partly comprised of fibers that form the hyperdirect pathway potentially useful as a target for PD by disrupting the synchrony of sensory motor networks (81).

**Figure 8:**
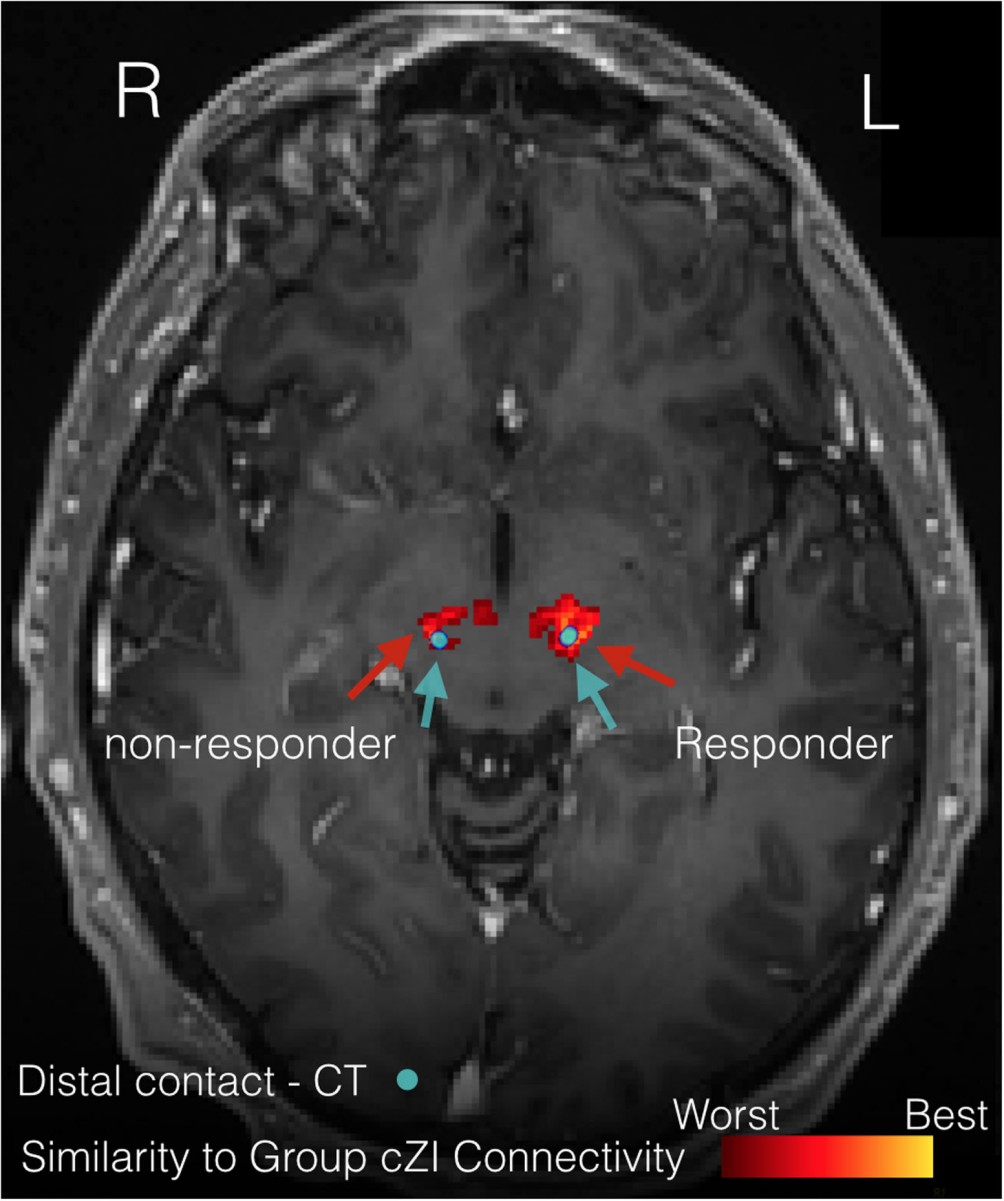
**Precision targeting at the individual patient level**. The figure illustrates the difference between patient-specific connectivity and group connectivity to guide targeting for a patient where the caudal zona incerta (cZi) has been targeted. The heat map shows where this patient’s connectivity is the closest match to the group connectivity average in patients having a good treatment response. The actual location of implants is shown on the postoperative computerized tomography (CT) scan (blue).

Figure 8 illustrates the benefits of precision targeting at the individual patient level for one of the cZI patients. In this patient, RMS difference with the average group connectivity from the cZI subjects is shown over the preoperative MRI (Figure 8, heat map), with the postoperative CT windowed to show only the implant (Figure 8, blue). The best match to the group average coincided almost exactly with the location of the implant on the right side and was approximately 1 mm lateral in the case of the left implant. Note that this surgery was planned in the conventional way without considering DWI. These results show that surgery could be aided by the use of DWI-derived connectivity, displayed in this heat map form which is easy to import into current surgical-planning software.

Deep brain stimulation for the treatment of psychiatric disorders has been gaining more attention since the FDA approved DBS (under a Humanitarian Device Exemption) for the treatment of OCD in 2009 based on evidence that DBS of the ventral internal capsule and ventral striatum may alleviate symptoms in intractable cases of this disorder (82, 83). Since then, DBS has been extensively studied for the treatment of a number of psychiatric disorders, such as MDD and addiction (84). Despite some progress, including insights gained from of a rich body of animal work on basic mechanisms of DBS (85), success in humans has been limited compared to movement disorders. This is primarily because the DBS targets for psychiatric disorders are often located in high order associate cortices where inter-individual variability is greater than for the phylogenetically older subcortical structures targeted in movement disorders. The structures targeted in movement disorders, such as the dorsolateral portion of the STN and the posterolateral GPi, have cortical connections that are very well conserved from patient to patient. By contrast, regions targeted in psychiatric disease, such as the NAc, have high inter-individual variability in their connectivity. Lehman and colleagues (86) demonstrate this clearly using the example of the vPFC, a regions with complex intrinsic functional organization and widespread connections with other cortical and subcortical regions. Their findings are based on conventional tracing techniques and 3D pathway reconstructions of the white matter connectomic fingerprint of the vPFC in the primate brain. In the preceding section on precision mapping, we discuss the same issue and demonstrate individual variability in vmPFC connectivity in healthy humans. The topographic organization of the efferent and afferent fibers to the vPFC suggests that variations in DBS electrode placement are likely to affect very different cortical and subcortical circuits and that only modulation of a selective subset of fibers may have therapeutic effects (72, 73). This suggests that in psychiatric disorders the ability to characterize individual differences in white matter networks may be even more important than in movement disorders. Thus, the patient-specific, atlas-free approach is applicable and perhaps essential to treatment of psychiatric disease with DBS.

**Figure 9:**
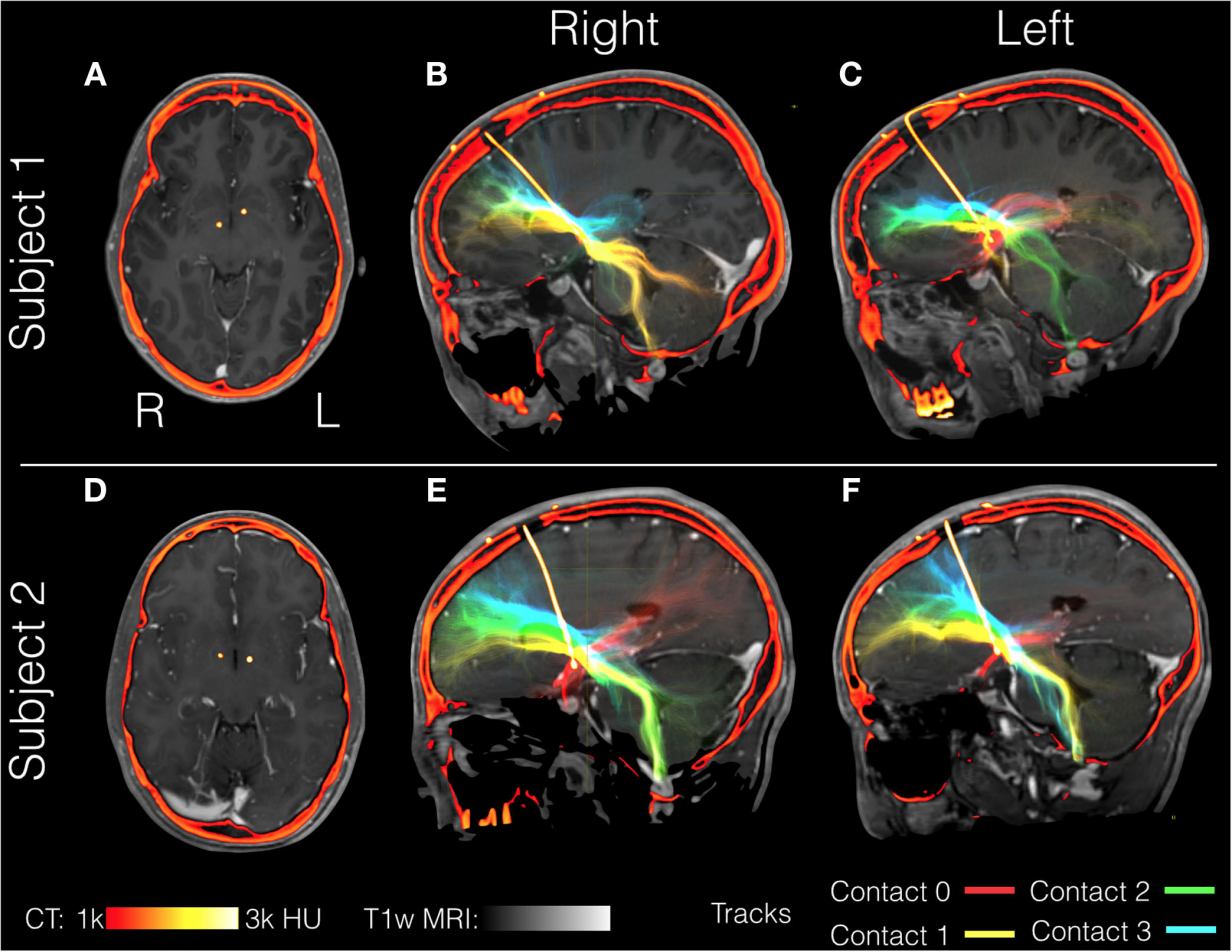
**Individual patient tractography based on electrode location in OCD**. Tractography from contacts in two patients with obsessive compulsive disorder (OCD), Subject 1 **(A–C)** and Subject 2 **(D–F)**. Fiber tracts are color-coded starting from the most distal: contact 0 (red), contact 1 (yellow), contact 2 (green), and contact 4 (blue). Structural T1-weighted MRI is shown in gray, with postoperative computerized tomography (CT) shown in hot colors depicting the skull and the electrode. Axial views show the location of the most distal electrodes **(A,C)** while the sagittal views show the entire electrode including the point it enters the burr hole at the top of the image **(B,C,E,F)**.

Here, we illustrate the use of precision targeting in two patients undergoing bilateral DBS of the ALIC for the treatment of OCD. Patients gave informed consent to participate in this study in accordance with a protocol approved by our institutional review board. Aside from the target selection, we followed the same imaging procedures, and data processing procedure outlined above for PD patients. OCD patients were implanted bilaterally with Medtronic model number 3391. This lead differed from the models used in the PD patients notably in that the electrode spacing is greater (4 vs. 1.5 mm in the 3387 or 0.5 mm in the 3389) and the contact size is larger (3 vs. 1.5 mm). Fiber tracking from all four contacts in both OCD patients is shown in Figure 9. The relative position of the most distal contact to the anterior commissure of each implant is depicted in the axial slices (Figures 9A,D). In Patient 1, the right implant from was placed more posterior than the left implant and both implants in Patient 2. Consequently, each contact interacts with a different pattern of tracks. In Patient 1, the right implant in all four contacts stimulates tracks with similar trajectories toward the frontal cortex and contacts 0 and 1 (the two most distal) show significant cerebellar components. In Patient 1, the left implant is placed within tracts that project to more inferior frontal lobe location than those in contact with the right implant. Contact 0 from the left implant lacks significant projections to the frontal lobe and instead projects toward the amygdala and temporal lobe, possibly tracing the amygdalofugal tract. In Patient 2, the most distal contacts in both leads show the same amygdala-temporal pattern as the left contact 0 from Patient 1. In Patient 2, the proximal contacts fan out toward the frontal lobe on both sides with the more proximal contacts projecting more superiorly. In these two patients, the contacts that showed the amygdala–temporal connectivity pattern (Patient 1: left contact 0; Patient 2: left and right contact 0) showed anxiety responses during programing. This suggests that in OCD, amygdalofugal involvement during DBS may be predictive of anxiety side effects (87).

At present, there is no clear consensus regarding the optimal DBS target for OCD (78–80). In the cases, we describe targeting the ALIC resulted in individual variability in the fibers passing through the DBS leads and these individual differences that may be important in surgical planning. Characterizing these connectivity differences and discovering correlates to treatment efficacy may point to a connectivity-based target independent from the current anatomical references.

## Conclusion and Future Directions

Precision medicine utilizes biological and other data to optimize and personalize treatment. It firmly places the individual patient at the heart of clinical practice and demands that technological developments are directed toward tailoring care to individual-specific variation. In the present paper, we highlight developments that aim to harness the power of neuroimaging for precision psychiatry. It is clear that the field is still in its infancy, and many challenges need to be addressed before the techniques described here are ready for deployment in routine clinical practice. Our quest for a better understanding of the mechanisms that lead to psychiatric disorders is essentially to find biological features that are informative in terms of diagnosis, prognosis, and treatment. Disruption in brain organization is the most proximal cause of psychiatric disorders and neuroimaging provides an invaluable tool for identifying and characterizing clinically informative features. Developments described here provide a roadmap for advancement starting from what is currently achievable.

## Author Contributions

All authors contributed to the writing of the manuscript and approved the final version. Drs RO, BK, and WG provided data on precision targeting in patients undergoing deep brain stimulation under their care. Dr. SF provided the data and analyses for the section on supervised classification based on functional imaging data. Dr. ES provided data and conducted analyses for the section of precision mapping.

## Conflict of Interest Statement

The authors declare that the research was conducted in the absence of any commercial or financial relationships that could be construed as a potential conflict of interest. The reviewer ND and handling Editor declared their shared affiliation, and the handling Editor states that the process nevertheless met the standards of a fair and objective review.

## Footnotes

^1^www.fil.ion.ucl.ac.uk/spm/software/spm8/

^2^www.mlnl.cs.ucl.ac.uk/pronto/

^3^http://surfer.nmr.mgh.harvard.edu/

^4^https://fsl.fmrib.ox.ac.uk/fsl/fslwiki/FLIRT

^5^https://github.com/MRtrix3/mrtrix3

## References

[1] Hyman SE. Can neuroscience be integrated into the DSM-V? Nat Rev Neurosci (2007) 8:725–32. 10.1038/nrn2218 17704814

[2] Wen W He Y Sachdev P. Structural brain networks and neuropsychiatric disorders. Curr Opin Psychiatry (2011) 24:219–25. 10.1097/YCO.0b013e32834591f8 21430538

[3] Greicius M. Resting-state functional connectivity in neuropsychiatric disorders. Curr Opin Neurol (2008) 21:424–30. 10.1097/WCO.0b013e328306f2c5 18607202

[4] Borgwardt S Fusar-Poli P. Third-generation neuroimaging in early schizophrenia: translating research evidence into clinical utility. Br J Psychiatry (2012) 200:270–2. 10.1192/bjp.bp.111.103234 22474231

[5] Bishop CM Pattern Pecognition and Machine Learning. New York: Springer (2006).

[6] Mourão-Miranda J Bokde AL Born C Hampel H Stetter M. Classifying brain states and determining the discriminating activation patterns: support vector machine on functional MRI data. Neuroimage (2005) 28:980–95. 10.1016/j.neuroimage.2005.06.070 16275139

[7] Orrù G Pettersson-Yeo W Marquand AF Sartori G Mechelli A. Using support vector machine to identify imaging biomarkers of neurological and psychiatric disease: a critical review. Neurosci Biobehav Rev (2012) 36:1140–52. 10.1016/j.neubiorev.2012.01.004 22305994

[8] Klöppel S Abdulkadir A Jack CR Jr Koutsouleris N Mourão-Miranda J Vemuri P. Diagnostic neuroimaging across diseases. Neuroimage (2012) 61:457–63. 10.1016/j.neuroimage.2011.11.002 22094642 PMC3420067

[9] Castellanos FX Di Martino A Craddock RC Mehta AD Milham MP. Clinical applications of the functional connectome. Neuroimage (2013) 80:527–40. 10.1016/j.neuroimage.2013.04.083 23631991 PMC3809093

[10] Wolfers T Buitelaar JK Beckmann CF Franke B Marquand AF. From estimating activation locality to predicting disorder: a review of pattern recognition for neuroimaging-based psychiatric diagnostics. Neurosci Biobehav Rev (2015) 57:328–49. 10.1016/j.neubiorev.2015.08.001 26254595

[11] World Health Organization. The Global Burden of Disease: 2004 Update. Geneva: World Health Organization (2008). Available from: http://www.who.int/healthinfo/global_burden_disease/GBD_report_2004update_full.pdf

[12] Rocha-Rego V Jogia J Marquand AF Mourao-Miranda J Simmons A Frangou S. Examination of the predictive value of structural magnetic resonance scans in bipolar disorder: a pattern classification approach. Psychol Med (2014) 44:519–32. 10.1017/S0033291713001013 23734914 PMC3880067

[13] Bansal R Staib LH Laine AF Hao X Xu D Liu J Anatomical brain images alone can accurately diagnose chronic neuropsychiatric illnesses. PLoS One (2012) 7(12):e50698. 10.1371/journal.pone.0050698 23236384 PMC3517530

[14] Schnack HG Nieuwenhuis M van Haren NE Abramovic L Scheewe TW Brouwer RM Can structural MRI aid in clinical classification? A machine learning study in two independent samples of patients with schizophrenia, bipolar disorder and healthy subjects. Neuroimage (2014) 84:299–306. 10.1016/j.neuroimage.2013.08.053 24004694

[15] Serpa MH Ou Y Schaufelberger MS Doshi J Ferreira LK Machado-Vieira R Neuroanatomical classification in a population-based sample of psychotic major depression and bipolar I disorder with 1 year of diagnostic stability. Biomed Res Int (2014) 2014:706157. 10.1155/2014/706157 24575411 PMC3915628

[16] Redlich R Almeida JJ Grotegerd D Opel N Kugel H Heindel W Brain morphometric biomarkers distinguishing unipolar and bipolar depression. A voxel-based morphometry-pattern classification approach. JAMA Psychiatry (2014) 71:1222–30. 10.1001/jamapsychiatry.2014.1100 25188810 PMC5538312

[17] Fung G Deng Y Zhao Q Li Z Qu M Li K Distinguishing bipolar and major depressive disorders by brain structural morphometry: a pilot study. BMC Psychiatry (2015) 15:298. 10.1186/s12888-015-0685-5 26590556 PMC4655080

[18] Costafreda SG Fu CH Picchioni M Toulopoulou T McDonald C Kravariti E Pattern of neural responses to verbal fluency shows diagnostic specificity for schizophrenia and bipolar disorder. BMC Psychiatry (2011) 11:18. 10.1186/1471-244X-11-18 21276242 PMC3042380

[19] Mourão-Miranda J Almeida JR Hassel S de Oliveira L Versace A Marquand AF Pattern recognition analyses of brain activation elicited by happy and neutral faces in unipolar and bipolar depression. Bipolar Disord (2012) 14:451–60. 10.1111/j.1399-5618.2012.01019.x 22631624 PMC3510302

[20] Grotegerd D Stuhrmann A Kugel H Schmidt S Redlich R Zwanzger P Amygdala excitability to subliminally presented emotional faces distinguishes unipolar and bipolar depression: an fMRI and pattern classification study. Hum Brain Mapp (2014) 35:2995–3007. 10.1002/hbm.22380 24038516 PMC6869222

[21] Rosa MJ Portugal L Hahn T Fallgatter AJ Garrido MI Shawe-Taylor J Sparse network-based models for patient classification using fMRI. Neuroimage (2015) 105:493–506. 10.1016/j.neuroimage.2014.11.021 25463459 PMC4275574

[22] Arribas JI Calhoun VD Adali T. Automatic Bayesian classification of healthy controls, bipolar disorder, and schizophrenia using intrinsic connectivity maps from FMRI data. IEEE Trans Biomed Eng (2010) 57:2850–60. 10.1109/TBME.2010.2080679 20876002 PMC2982883

[23] Frangou S Dima D Jogia J Towards person-centered neuroimaging markers for resilience and vulnerability in bipolar disorder. Neuroimage (2016). 10.1016/j.neuroimage.2016.08.066 PMC5555631 27622393

[24] Shih RA Belmonte PL Zandi PP. A review of the evidence from family, twin and adoption studies for a genetic contribution to adult psychiatric disorders. Int Rev Psychiatry (2004) 16:260–83. 10.1080/09540260400014401 16194760

[25] Ketter TA. Nosology, diagnostic challenges, and unmet needs in managing bipolar disorder. J Clin Psychiatry (2010) 71(10):e27. 10.4088/JCP.8125tx12c 21062613

[26] Judd LL Akiskal HS Schettler PJ Endicott J Leon AC Solomon DA Psychosocial disability in the course of bipolar I and II disorders: a prospective, comparative, longitudinal study. Arch Gen Psychiatry (2005) 62:1322–30. 10.1001/archpsyc.62.12.1322 16330720

[27] Hirschfeld RM Differential diagnosis of bipolar disorder and major depressive disorder. J Affect Disord (2014) 169(Suppl 1):12–6. 10.1016/S0165-0327(14)70004-7 25533909

[28] Mitchell PB Goodwin GM Johnson GF Hirschfeld RM. Diagnostic guidelines for bipolar depression: a probabilistic approach. Bipolar Disord (2008) 10:144–52. 10.1111/j.1399-5618.2007.00559.x 18199233

[29] Mitchell PB Frankland A Hadzi-Pavlovic D Roberts G Corry J Wright A Comparison of depressive episodes in bipolar disorder and in major depressive disorder within bipolar disorder pedigrees. Br J Psychiatry (2011) 199:303–9. 10.1192/bjp.bp.110.088823 21508436

[30] Forty L Jones L Jones I Smith DJ Caesar S Fraser C Polarity at illness onset in bipolar I disorder and clinical course of illness. Bipolar Disord (2009) 11:82–8. 10.1111/j.1399-5618.2008.00654.x 19133970

[31] Duffy A Jones S Goodday S Bentall R. Candidate risks indicators for bipolar disorder: early intervention opportunities in high-risk youth. Int J Neuropsychopharmacol (2015). 10.1093/ijnp/pyv071 26116493 PMC4772266

[32] Gevins A Smith ME. Neurophysiological measures of working memory and individual differences in cognitive ability and cognitive style. Cereb Cortex (2000) 10:829–39. 10.1093/cercor/10.9.829 10982744

[33] Frangou S. Risk and resilience in bipolar disorder: rationale and design of the vulnerability to bipolar disorders study (VIBES). Biochem Soc Trans (2009) 37:1085–9. 10.1042/BST0371085 19754457

[34] Kempton MJ Haldane M Jogia J Grasby PM Collier D Frangou S. Dissociable brain structural changes associated with predisposition, resilience, and disease expression in bipolar disorder. J Neurosci (2009) 29:10863–8. 10.1523/JNEUROSCI.2204-09.2009 19726644 PMC6665540

[35] Kempton MJ Ruberto G Vassos E Tatarelli R Girardi P Collier D Effects of the CACNA1C risk allele for bipolar disorder on cerebral gray matter volume in healthy individuals. Am J Psychiatry (2009) 166:1413–45. 10.1176/appi.ajp.2009.09050680 19952088

[36] Walterfang M Wood AG Barton S Velakoulis D Chen J Reutens DC Corpus callosum size and shape alterations in individuals with bipolar disorder and their first-degree relatives. Prog Neuropsychopharmacol Biol Psychiatry (2009) 33:1050–7. 10.1016/j.pnpbp.2009.05.019 19500633

[37] Takahashi T Walterfang M Wood SJ Kempton MJ Jogia J Lorenzetti V Pituitary volume in patients with bipolar disorder and their first-degree relatives. J Affect Disord (2010) 124:256–61. 10.1016/j.jad.2009.12.002 20022640

[38] Forcada I Papachristou E Mur M Christodoulou T Jogia J Reichenberg A The impact of general intellectual ability and white matter volume on the functional outcome of patients with bipolar disorder and their relatives. J Affect Disord (2011) 130:413–20. 10.1016/j.jad.2010.10.048 21112093

[39] Lelli-Chiesa G Kempton MJ Jogia J Tatarelli R Girardi P Powell J The impact of the Val158Met catechol-O-methyltransferase genotype on neural correlates of sad facial affect processing in patients with bipolar disorder and their relatives. Psychol Med (2011) 41:779–88. 10.1017/S0033291710001431 20667170

[40] Perrier E Pompei F Ruberto G Vassos E Collier D Frangou S. Initial evidence for the role of CACNA1C on subcortical brain morphology in patients with bipolar disorder. Eur Psychiatry (2011) 26:135–7. 10.1016/j.eurpsy.2010.10.004 21292451

[41] Pompei F Dima D Rubia K Kumari V Frangou S. Dissociable functional connectivity changes during the Stroop task relating to risk, resilience and disease expression in bipolar disorder. Neuroimage (2011) 57:576–82. 10.1016/j.neuroimage.2011.04.055 21570470

[42] Pompei F Jogia J Tatarelli R Girardi P Rubia K Kumari V Familial and disease specific abnormalities in the neural correlates of the Stroop task in bipolar disorder. Neuroimage (2011) 56:1677–84. 10.1016/j.neuroimage.2011.02.052 21352930

[43] Ruberto G Vassos E Lewis CM Tatarelli R Girardi P Collier D The cognitive impact of the ANK3 risk variant for bipolar disorder: initial evidence of selectivity to signal detection during sustained attention. PLoS One (2011) 6(1):e16671. 10.1371/journal.pone.0016671 21304963 PMC3031622

[44] Jogia J Ruberto G Lelli-Chiesa G Vassos E Maierú M Tatarelli R The impact of the CACNA1C gene polymorphism on frontolimbic function in bipolar disorder. Mol Psychiatry (2011) 16:1070–1. 10.1038/mp.2011.49 21519340

[45] Jogia J Dima D Kumari V Frangou S. Frontopolar cortical inefficiency may underpin reward and working memory dysfunction in bipolar disorder. World J Biol Psychiatry (2012) 13:605–15. 10.3109/15622975.2011.585662 21812622

[46] Dima D Jogia J Collier D Vassos E Burdick KE Frangou S. Independent modulation of engagement and connectivity of the facial network during affect processing by CACNA1C and ANK3 risk genes for bipolar disorder. JAMA Psychiatry (2013) 70:1303–11. 10.1001/jamapsychiatry.2013.2099 24108394

[47] Delvecchio G Dima D Frangou S. The effect of ANK3 bipolar-risk polymorphisms on the working memory circuitry differs between loci and according to risk-status for bipolar disorder. Am J Med Genet B Neuropsychiatr Genet (2015) 168B:188–96. 10.1002/ajmg.b.32294 25711502

[48] Dima D Roberts RE Frangou S. Connectomic markers of disease expression, genetic risk and resilience in bipolar disorder. Transl Psychiatry (2016) 6:e706. 10.1038/tp.2015.193 26731443 PMC5068872

[49] Schrouff J Rosa MJ Rondina JM Marquand AF Chu C Ashburner J PRoNTo: pattern recognition for neuroimaging toolbox. Neuroinformatics (2013) 11:319–37. 10.1007/s12021-013-9178-1 23417655 PMC3722452

[50] Huisman TA. Diffusion-weighted and diffusion tensor imaging of the brain, made easy. Cancer Imaging (2010) 10:163–71. 10.1102/1470-7330.2010.9023 20880787 PMC2967146

[51] Frost MA Goebel R Measuring structural-functional correspondence: spatial variability of specialised brain regions after macro-anatomical alignment. Neuroimage (2012) 59:1369–81. 10.1016/j.neuroimage.2011.08.035 21875671

[52] Wang D Buckner RL Fox MD Holt DJ Holmes AJ Stoecklein S Parcellating cortical functional networks in individuals. Nat Neurosci (2015) 18:1853–60. 10.1038/nn.4164 26551545 PMC4661084

[53] Sohn WS Yoo K Lee YB Seo SW Na DL Jeong Y. Influence of ROI selection on resting state functional connectivity: an individualized approach for resting state fMRI analysis. Front Neurosci (2015) 9:280. 10.3389/fnins.2015.00280 26321904 PMC4531302

[54] Passingham RE Stephan KE Kötter R The anatomical basis of functional localization in the cortex. Nat Rev Neurosci (2002) 3:606–16. 10.1038/nrn893 12154362

[55] Jbabdi S Lehman JF Haber SN Behrens TE Human and monkey ventral prefrontal fibers use the same organizational principles to reach their targets: tracing versus tractography. J Neurosci (2013) 33:3190–201. 10.1523/JNEUROSCI.2457-12.2013 23407972 PMC3602794

[56] Rigoard P Buffenoir K Jaafari N Giot JP Houeto JL Mertens P The accumbofrontal fasciculus in the human brain: a microsurgical anatomical study. Neurosurgery (2011) 68:1102–11. 10.1227/NEU.0b013e3182098e48 21242843

[57] Behrens TE Berg HJ Jbabdi S Rushworth MF Woolrich MW. Probabilistic diffusion tractography with multiple fibre orientations: what can we gain? Neuroimage (2007) 34:144–55. 10.1016/j.neuroimage.2006.09.018 17070705 PMC7116582

[58] Miranda-Dominguez O Mills BD Grayson D Woodall A Grant KA Kroenke CD Bridging the gap between the human and macaque connectome: a quantitative comparison of global interspecies structure-function relationships and network topology. J Neurosci (2014) 34:5552–63. 10.1523/JNEUROSCI.4229-13.2014 24741045 PMC3988411

[59] Osher D Tobyne S Congden K Michalka S Somers D. Structural and functional connectivity of visual and auditory attentional networks: insights from the Human Connectome Project. J Vis (2015) 15:223. 10.1167/15.12.22326325911

[60] Ullo S Nieus TR Sona D Maccione A Berdondini L Murino V. Functional connectivity estimation over large networks at cellular resolution based on electrophysiological recordings and structural prior. Front Neuroanat (2014) 8:137. 10.3389/fnana.2014.00137 25477790 PMC4238367

[61] DeLong MR Wichmann T. Basal ganglia circuits as targets for neuromodulation in Parkinson disease. JAMA Neurol (2015) 72:1354–60. 10.1001/jamaneurol.2015.2397 26409114

[62] Collomb-Clerc A Welter ML. Effects of deep brain stimulation on balance and gait in patients with Parkinson’s disease: a systematic neurophysiological review. Neurophysiol Clin (2015) 45:371–88. 10.1016/j.neucli.2015.07.001 26319759

[63] Combs HL Folley BS Berry DT Segerstrom SC Han DY Anderson-Mooney AJ Cognition and depression following deep brain stimulation of the subthalamic nucleus and globus pallidus pars internus in Parkinson’s disease: a meta-analysis. Neuropsychol Rev (2015) 25:439–54. 10.1007/s11065-015-9302-0 26459361

[64] Blomstedt P Fytagoridis A Åström M Linder J Forsgren L Hariz MI. Unilateral caudal zona incerta deep brain stimulation for parkinsonian tremor. Parkinsonism Relat Disord (2012) 18:1062–76. 10.1016/j.parkreldis.2012.05.024 22709794

[65] Ranck JB Jr. Which elements are excited in electrical stimulation of mammalian central nervous system: a review. Brain Res (1975) 98:414–40. 1102064 10.1016/0006-8993(75)90364-9

[66] Grill WM Mclntyre CC Extracellular excitation of central neurons: implications for the mechanisms of deep brain stimulation. Thalamus Relat Syst (2001) 1:269–77. 10.1016/S1472-9288(01)00025-5

[67] Herrington TM Cheng JJ Eskandar EN Mechanisms of deep brain stimulation. J Neurophysiol (2015) 115:19–38. 10.1152/jn.00281.2015 26510756 PMC4760496

[68] Gradinaru V Mogri M Thompson KR Henderson JM Deisseroth K. Optical deconstruction of parkinsonian neural circuitry. Science (2009) 324:354–9. 10.1126/science.1167093 19299587 PMC6744370

[69] Torres CV Manzanares R Sola RG. Integrating diffusion tensor imaging-based tractography into deep brain stimulation surgery: a review of the literature. Stereotact Funct Neurosurg (2014) 92:282–90. 10.1159/000362937 25248076

[70] Coenen VA Allert N Paus S Kronenbürger M Urbach H Mädler B. Modulation of the cerebello-thalamo-cortical network in thalamic deep brain stimulation for tremor: a diffusion tensor imaging study. Neurosurgery (2014) 75:657–69. 10.1227/NEU.0000000000000540 25161000

[71] Riva-Posse P Choi KS Holtzheimer PE McIntyre CC Gross RE Chaturvedi A Defining critical white matter pathways mediating successful subcallosal cingulate deep brain stimulation for treatment-resistant depression. Biol Psychiatry (2014) 76:963–9. 10.1016/j.biopsych.2014.03.029 24832866 PMC4487804

[72] Hartmann CJ Lujan JL Chaturvedi A Goodman WK Okun MS McIntyre CC Tractography activation patterns in dorsolateral prefrontal cortex suggest better clinical responses in OCD DBS. Front Neurosci (2016) 9:519. 10.3389/fnins.2015.00519 26834544 PMC4717315

[73] Makris N Rathi Y Mouradian P Bonmassar G Papadimitriou G Ing WI Variability and anatomical specificity of the orbitofrontothalamic fibers of passage in the ventral capsule/ventral striatum (VC/VS): precision care for patient-specific tractography-guided targeting of deep brain stimulation (DBS) in obsessive compulsive disorder (OCD). Brain Imaging Behav (2015) 1–14. 10.1007/s11682-015-9462-9 26518214 PMC4851930

[74] Jaeger D Kita H. Functional connectivity and integrative properties of globus pallidus neurons. Neuroscience (2011) 198:44–53. 10.1016/j.neuroscience.2011.07.050 21835227 PMC3221766

[75] Rozanski VE Vollmar C Cunha JP Tafula SM Ahmadi SA Patzig M Connectivity patterns of pallidal DBS electrodes in focal dystonia: a diffusion tensor tractography study. Neuroimage (2014) 84:435–42. 10.1016/j.neuroimage.2013.09.009 24045076

[76] van den Heuvel MP Sporns O Network hubs in the human brain. Trends Cogn Sci (2013) 17:683–96. 10.1016/j.tics.2013.09.012 24231140

[77] Li W Wu B Liu C. Quantitative susceptibility mapping of human brain reflects spatial variation in tissue composition. Neuroimage (2011) 55:1645–56. 10.1016/j.neuroimage.2010.11.088 21224002 PMC3062654

[78] Tournier JD Calamante F Gadian DG Connelly A. Direct estimation of the fiber orientation density function from diffusion-weighted MRI data using spherical deconvolution. Neuroimage (2004) 23:1176–85. 10.1016/j.neuroimage.2004.07.037 15528117

[79] Tournier JD Calamante F Connelly A. Robust determination of the fibre orientation distribution in diffusion MRI: non-negativity constrained super-resolved spherical deconvolution. Neuroimage (2007) 35:1459–72. 10.1016/j.neuroimage.2007.02.016 17379540

[80] Smith RE Tournier JD Calamante F Connelly A. Anatomically-constrained tractography: improved diffusion MRI streamlines tractography through effective use of anatomical information. Neuroimage (2012) 62:1924–38. 10.1016/j.neuroimage.2012.06.005 22705374

[81] Whitmer D De Solages C Hill D Yu H Henderson JM Bronte-Stewart H. High frequency deep brain stimulation attenuates subthalamic and cortical rhythms in Parkinson’s disease. Front Hum Neurosci (2012) 4(6):155. 10.3389/fnhum.2012.00155 22675296 PMC3366347

[82] Greenberg BD Gabriels LA Malone DA Jr Rezai AR Friehs GM Okun MS Deep brain stimulation of the ventral internal capsule/ventral striatum for obsessive-compulsive disorder: worldwide experience. Mol Psychiatry (2010) 15:64–79. 10.1038/mp.2008.55 18490925 PMC3790898

[83] Castle D Bosanac P Rossell S. Treating OCD: what to do when first-line therapies fail. Australas Psychiatry (2015) 23:350–3. 10.1177/1039856215590027 26104775

[84] Cleary DR Ozpinar A Raslan AM Ko AL. Deep brain stimulation for psychiatric disorders: where we are now. Neurosurg Focus (2015) 38:E2. 10.3171/2015.3.FOCUS1546 26030702

[85] Hamani C Temel Y. Deep brain stimulation for psychiatric disease: contributions and validity of animal models. Sci Transl Med (2012) 4:142rv8. 10.1126/scitranslmed.3003722 22786683

[86] Lehman JF Greenberg BD McIntyre CC Rasmussen SA Haber SN. Rules ventral prefrontal cortical axons use to reach their targets: implications for diffusion tensor imaging tractography and deep brain stimulation for psychiatric illness. J Neurosci (2011) 31:10392–402. 10.1523/JNEUROSCI.0595-11.2011 21753016 PMC3445013

[87] Shapira NA Okun MS Wint D Foote KD Byars AJ Bowers D Panic and fear induced by deep brain stimulation. J Neurol Neurosurg Psychiatry (2006) 77:410–2. 10.1136/jnnp.2005.069906 16484657 PMC2077710

